# Application of an active middle ear implant in congenital middle ear malformations: A contemporary review

**DOI:** 10.1016/j.bjorl.2025.101562

**Published:** 2025-02-05

**Authors:** Vagner Antonio Rodrigues da Silva, Henrique Furlan Pauna, Guilherme Correa Guimarães, Joel Lavinsky, Thomas E. Linder, Arthur Menino Castilho

**Affiliations:** a Faculdade de Ciências Médicas da Universidade Estadual de Campinas (UNICAMP), Departamento de Otorrinolaringologia e Cirurgia de Cabeça e Pescoço, Campinas, SP, Brazil; b Hospital Universitário Cajuru, Departamento de Otorrinolaringologia, Curitiba, PR, Brazil; c Universidade Federal do Rio Grande do Sul (UFRGS), Departamento de Cirurgia, Porto Alegre, RS, Brazil; d Hals-, Nasen-, Ohrenklinik, Luzerner Kantonsspital, Luzern, Switzerland

**Keywords:** Hearing loss, External ear malformation, Ear surgery, Hearing aids, Middle ear implants

## Abstract

**Objective:**

To evaluate hearing outcomes and postoperative complications among patients with middle and external ear malformations undergoing active middle ear implantation with Vibrant Soundbridge® (VSB).

**Methods:**

Review of the literature. Studies published in English, Portuguese, or Spanish at the following databases: PubMed, MEDLINE, Scopus, Web of Science, EMBASE, and Cochrane Library were searched. The search strategy yielded a total of 141 potentially relevant studies. Of these, ten were included in this analysis.

**Results:**

The mean preoperative air conduction threshold was 66.7 ± 6.2 dB. The mean air-bone gap was 46 ± 7.7 dB. VSB implantation resulted in mean hearing gain of 40.5 ± 7.1 dB in the air-conduction thresholds among the evaluated frequencies. The speech recognition index if the Floating Mass Transducer (FMT) was placed in the short process was 86.0% ± 9.6%, with significant difference when compared to long process coupling (*p* = 0.035) and the round window coupling (*p* =  0.048).

**Conclusion:**

Bone conduction thresholds did not worsen in any of the studies included in the present review. VSB implantation resulted in a mean hearing gain of 40 dB at air conduction thresholds.

## Highlights

• External ear malformations frequently lead to conductive hearing loss.
• The Jahrsdoerfer score is used to qualify patients for corrective surgery.
• Patients presented improvement of bone and air-conduction thresholds.

## Introduction

Microtia and congenital aural atresia are distinct types of ear malformations that occur in one every 10,000–20,000 births, approximately (male:female ratio, 2.5:1). Conductive hearing loss is the most frequent auditory deficit in these patients, as the inner ear and its function are often preserved.^1^

The type of treatment ‒ surgical or non-surgical ‒ depends on several factors. Surgical procedures are indicated for patients with minor malformations and favorable anatomy of the temporal bone.^2^ Otherwise, alternative solutions for hearing rehabilitation, such as implantable hearing aids, are available.^3^

The ultimate goal of surgical treatment is to restore hearing and create an External Auditory Canal (EAC) that remains patent and free of infection.^4^ However, postoperative complications, such as EAC restenosis, can hinder optimal functional results. In addition, peripheral facial palsy, graft lateralization, and chronic infection may also occur.^5^, ^6^

The Jahrsdoerfer grading system is used to classify patients preoperatively.^7^ Patients receive a score of 1–10 according to physical examination findings and temporal bone Computed Tomography (CT) scan results. The final score indicates the degree of success that can be expected from the surgical treatment: a score of 5 or less disqualifies the patient for surgery, as the risks of the procedure outweigh the potential benefits.^2^

The expected level of post-surgical auditory functional improvement varies depending on the severity of the middle ear malformation and the presence of associated inner ear malformations.

For children under five years of age, bone-conduction implants with elastic bands are often used for their conductive hearing loss. From this age onward, bone- conduction implants or active middle ear implants can be surgically implanted.^8^ One such device, the Vibrant Soundbridge® (VSB; Med-El®, Innsbruck, Austria), is used to treat patients aged three years and older with middle ear malformations.^9^

The implanted portion of the VSB, the vibrating ossicular replacement prosthesis, comprises of a receiving coil, a conductor link, and the Floating Mass Transducer (FMT).^10^, ^11^ Previously, the FMT was placed only on the long process of the incus. The frequency response of the VSB ranges from 100 to 10,000 Hz with a coupling‐dependent average gain of 30–55 dB.^10^ However, new couplers allow the FMT to be placed on the short process of the incus, stapes superstructure, and round window as well.^12^, ^13^ The safety of the VSB and the efficiency of the various coupling modalities within the indication range have been established over many years. In the present study, authors focused to present a contemporary review of active middle ear implants in congenital external and middle ear malfomations. To our knowledge, this is the first review of the literature comparing the audiological results after VSB surgery.

The objective of the presented study was to evaluate hearing outcomes and postoperative complications among patients with middle and external ear malformations undergoing VSB surgery. Additionally, we compared the results of FMT placement in the round window with other FMT coupling sites (such as stapes superstructure, stapes footplate, long process of the incus, short process of the incus).

## Methods

This review was conducted in accordance with the Preferred Reporting Items for Systematic Reviews and Meta-analyses (PRISMA) guidelines^14^ and the current recommendations of the Cochrane Collaboration. Additionally, this review is registered in PROSPERO (ID #CRD42024510945).

Previous studies published in English, Portuguese, or Spanish that assessed the effectiveness and risks of using the VSB in congenital aural atresia at various coupling sites (incus, stapes, and round window) were selected. The databases searched included PubMed, Scopus, Web of Science, EMBASE, and the Cochrane Library. No restrictions were placed on publication dates.

In all databases, the following search terms (MeSH terms) were used: “Congenital aural atresia” OR “Aural Atresia, Congenital” AND “Vibrant Soundbridge”. The complete search strategy is provided as supplementary material. The reviewers used two reference managers EndNote X8 and Rayyan QCRI to consolidate the data extracted from the databases.

The following parameters were evaluated in each study: sample size, pure-tone threshold detection, speech audiometry, Jahrsdoerfer score, surgical complications, and coupling site of the FMT. Exclusion criteria were review articles without clinical cases, microtia without external ear canal atresia, experimental cadaveric studies, and single case reports.

Unrelated titles and duplicated studies were removed. Two independent reviewers screened the titles and abstracts, and any disagreement was resolved by consulting a third reviewer for arbitration. Only data reported in the body of the article, in tables or that could be accurately calculated from graphs, numbers, or raw data sets were used.

The quality of studies was assessed using the Oxford Center for Evidence‐ Based Medicine Levels of Evidence. Studies were included if the evaluation was based on the mean air- and bone-conduction thresholds, considering the frequencies of 500 to 4,000 Hz, before and after the surgery. The air-bone gap (difference between air- and bone-conduction thresholds) was calculated by subtracting the respective thresholds. The remaining studies were excluded. The Jahrsdoerfer grading system was used in the selection of operated patients.^5^

All analyses were performed using SPSS, version 22.0. Results are presented as the mean ± Standard Deviation (SD). For all tests, a *p-*value lower than 0.05 was considered significant.

## Results

**Fig. 1:**
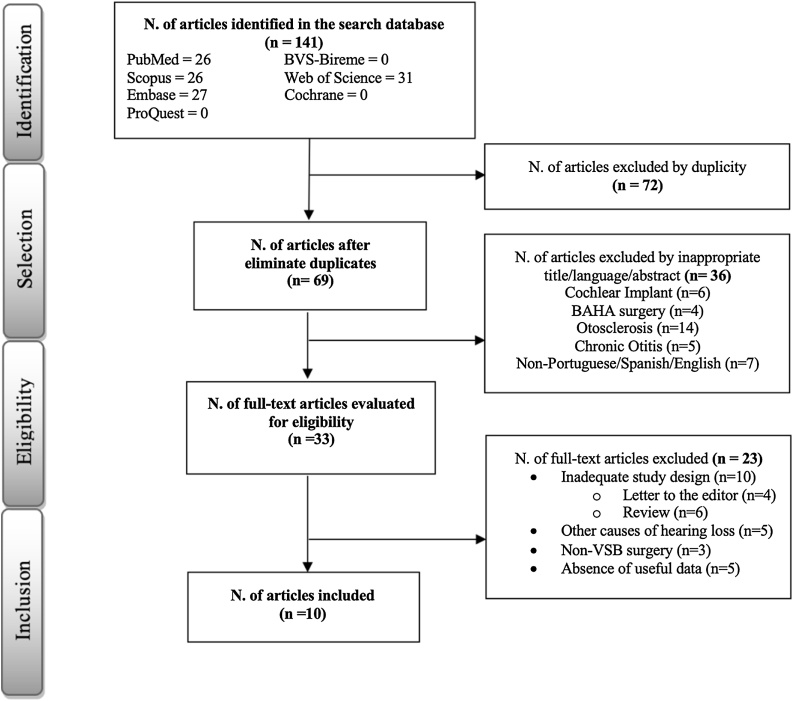
Identification of studies - databases and registers.

Initial search yielded 141 potentially relevant studies. After the removal of duplicates, 69 remained for the screening of titles and abstracts. After reviewing the titles and abstracts, 33 studies were selected for full-text reading. From the 33 studies, 10 were included in this analysis (four prospective and six retrospective studies). Fig. 1 provides an overview of the study selection process according to the PRISMA guidelines.

The mean age of the children included in most of the studies was 16 ± 7.2 years (range, 3–44 years). The Jahrsdoerfer score ranged from 2 to 10, with a mean score of 7.2 ± 1.8 points (Table 1). Six patients in the study by Thomas et al. were excluded from the final analysis as they had acquired stenosis rather than middle ear malformations.^15^ In all studies, individual data was available for all patients with middle ear atresia.

**Table 1:** Demographic characteristics of included studies (n = 10).

First author	Nº of patients	Mean age (years)	Mean Jahrsdoerfer score
Cellerier ^18^	3	11.6	7.6
Colletti ^17^	12	10.2	5.5
Lesinkas ^19^	3	11.6	4
Clarós ^20^	14	13.7	7.8
Roman ^8^	12	9.7	9.8
Brito ^26^	12	20.1	8
Thomas ^15^	7	11.7	8
Verharert ^7^	2	28	7
Wang ^43^	4	8	5
Zernotti ^16^	12	22.4	6.2
**Total**	**81**	**16.5 (6.7)**	**7.2 (1.8)**

Standard deviation in parentheses.

The most common coupling site was the round window, followed by the stapes, short process of the incus, oval window, and long process of the incus (Table 2). For patients with higher Jahrsdoerfer scores (mean of 9.3 ± 0.9 points), the long process of the incus was the most used coupling site. Conversely, the round window was preferred for patients with lower scores (mean of 5.9 ± 1.5 points).

**Table 2:** Mean postoperative results with the Vibrant Soundbridge® (VSB) on according to coupling site and Jahrsdoerfer score.

Coupling site (N)	Jahrsdoerfer score	Pre ACT (dB)	Pre ABG	Pre SRI (dB)	Post ACT^a^ (dB)	Post ABG	Post SRI^a^ (dB)
Incus ‒ long process ^11^	9.3	65	40.2	34.90	25.41	14.79	100
Incus ‒ short process ^11^	7.8	64.5	57.2	39.18	24	33.2	86
Oval window ^15^	7.4	75	42.2	17.21	33.85	8.85	96.92
Stapes ^24^	7.1	63.43	46.7	42.08	26.38	20.32	91
Round window ^20^	5.9	75	43.9	12.74	30.31	13.59	98
**Total (SD)**	**7.29 (1.82)**	**66.78 (6.22)**	**46.07 (7.73)**	**28.48 (19.58)**	**28.10 (7.87)**	**17.72 (7.57)**	**92.8 (7.98)**

ABG, Air-Bone Gap; ACT, Air Conduction Threshold; N, Number of each coupling site; Pre, Preoperative; Post, Postoperative; SD, Standard Deviation; SRI, Speech Recognition index.

a VSB on.

The mean preoperative air-conduction threshold was 66.7 ± 6.2 dB and the mean air-bone gap was 46.0 ± 7.7 dB. Hearing loss among patients was predominantly conductive. In all included studies, bone conduction threshold was 25 dB or better, except for Roman et al., which reported a mean bone-conduction threshold of 27 dB.^8^

Comparative analysis of the pre-operative mean air-conduction thresholds within the frequencies of 500 Hz to 4.000 Hz versus post-operatively (with the VSB) showed an average improvement of 40.5 ± 7.12 dB in hearing thresholds. The round window coupling achieved the best air-conduction threshold gain (44.6 ± 7.5 dB), followed by the oval window coupling site (41.1 ± 5.3 dB).

The authors of all included studies reported no significant variability in bone- conduction thresholds. Zernotti et al.^16^ reported a mean bone-conduction hearing loss of 5 dB, mainly in round and oval window coupling.

Some studies reported postoperative complications. Those included: one case of facial palsy (Thomas et al.)^15^ which improved with medical treatment in approximately six days; one case of hematoma (Zernotti et al.),^16^ which was treated and resolved with compressive dressing; and two cases of mild dizziness on the first postoperative day (Colletti et al.).^17^

## Discussion

Congenital aural atresia leads to aesthetic problems and hearing deficits. Atresiaplasty can be a challenging surgery, often yielding poor functional results along with a high rate of restenosis and complications. For this reason, the use of implantable hearing aids have been becoming the standard of care for patients who fulfill specific surgical indication criteria.^5^

The present study outlined data from patients undergoing VSB surgery, and no comparisons were made with other devices. Colletti et al.^17^ placed the FMT at the round window among four children under three years of age. In their study, only two children (under 4 years of age) had their VSB implant coupled to the short process of the incus.^18^

Regarding associated inner ear malformations, dysplasia of the semicircular canals (especially the lateral semicircular canal) was the most commonly associated with external auditory canal atresia.^19^, ^20^ Lower Jahrsdoerfer scores increased the risk of abnormalities of the oval and round windows, making the use of VSB implants more difficult. Abnormalities have been reported in approximately 7% of cases in the round window and in 21% in the oval window.^21^

### Audiological results

Different couplers have been developed over time, simplifying the surgical procedure, especially when the FMT is placed on the short process of the incus.^11^ Vyskocil et al.^22^ and Sprinzl et al.^23^ found an audiometric gain of 30–58 dB in mixed or conductive hearing loss compared to the audiometric gain of 23–30 dB in sensorineural hearing loss, regardless of FMT placement site. This difference is essentially due to the mechanical action of FMT.^4^

The improvements in air-conduction thresholds were considered satisfactory in all the FMT coupling sites. The round window coupling achieved the highest post-surgical mean threshold gain and the stapes superstructure coupling had the lowest. Patients who had their VSB placed int the long process of the incus achieved the best speech recognition index, whereas the short process of the incus placement associated with the lowest scores in speech recognition index.

The retrospective analysis also demonstrated the performance of the FMT coupling modalities was similar for patients with mild-to-moderate hearing loss and bone-conduction hearing thresholds better than 50 dB HL. The middle ear condition strongly influences the choice of coupling technique, and the decision is often based on anatomical findings during surgery.

### Long process of the incus

The VSB was initially designed for coupling to the long process of the incus in patients with sensorineural hearing loss.^24^ There are challenges associated with performing long process vibroplasty, which requires a wide posterior tympanotomy to place the FMT properly and to fasten the clip accurately. Grégoire et al.^24^ implanted the FMT in 46 patients on the long process of the incus. They observed significant hearing gains, with no major complications, and noted no significant decrease in hearing compared to the contralateral ear.

In patients with higher Jahrsdoerfer scores, the implants were preferably placed on the long process of the incus. Patients had the best speech recognition index, satisfactory hearing gain, and median air-conduction thresholds near normality.

### Short process of the incus

The incus short process-coupler vibroplasty is performed without a posterior tympanotomy and only requires a mastoidectomy with wide epitympanotomy for FMT attachment, reducing the potential risk for facial nerve injury and surgical time.^25^ Lee et al.^11^ showed that short process vibroplasty could improve hearing gain and word recognition score compared to long process vibroplasty, except at high frequencies. Brito et al.^26^ placed the FMT at the short process of the incus, even in cases with a Jahrsdoerfer score of 5 points.

The hearing gain of air-conduction thresholds with the short process coupler was similar to the long process placement. The speech recognition index was the worst, significantly different from the long process and round window coupling.

### Oval window

The oval window niche procedure was described by authors as less complex to perform than round window placement.^27^ Canale et al.^28^ had similar audiological results with both round window and oval window coupling. Clarós et al.^20^ chose the oval window in most cases and observed best amplification (at 500 Hz) with oval window coupler.

There was no significant difference between oval window and short process of the incus coupling, but there was significant difference between oval window and stapes coupling. Considering the lower risk of complications, it is preferable to couple the FMT at the short process of the incus rather than coupling in oval window.^29^

### Stapes

A previous study on the human temporal bone model examined the mass loading effects on ossicles. The study revealed that the stapes footplate decreased in response to the increased weight, and the effect was more prominent at high frequencies.^30^ Other experimental studies demonstrated no significant changes below 900 Hz, regardless of the weight of the mass.^31^, ^32^, ^33^, ^34^ In most cases, the stapes was the site of choice for FMT coupling.

Stapes coupling had the worst hearing gain in air-conduction thresholds when compared to other coupling sites. When the FMT was coupled at the stapes superstructure, the speech recognition index was slightly better than the short process coupling. Perhaps it has occurred because the VORP 502 (Vibrant Soundbridge® Vibrating Ossicular Prosthesis, Med-El®, Innsbruck, Austria) was used rather the third generation (VORP 503).^3^, ^8^, ^26^ Despite that, the audiological results are satisfactory.

### Round window

The VSB was initially developed for sensorineural hearing loss, coupled to the long process of the incus. In 2006, a case series placing the VSB in the round window was described in patients with mixed hearing loss. The round window vibroplasty was performed with the FMT placed directly onto the round window membrane to directly deliver vibrational energy to the scala tympani.^35^ The use of a specific titanium coupler should provide a better connection by adapting the FMT to the smaller window and simplifying surgical procedure by reducing the drilling effort at the round window niche.

There is a higher effective gain for round window coupling. Positioning the FMT in the round window without a coupler can give unsatisfactory results, particularly at lower frequencies. In patients with mixed or conductive hearing loss, who needs a low- frequency amplification, the oval window approach should be preferred if round and oval windows are accessible. The anatomy of round window is variable, so the coupling can be less well-defined. The use of a round window coupler can reduce the variability in the results.^36^

Round window atresia can be a problem in few cases and requires drilling to access the scala tympani, which increases the risk of sensorineural hearing loss. It might be suggested to use piezoelectric drills to reduce this risk.^37^, ^38^ In our study, none of the patients who received a VSB implant into the round window had sensorineural hearing loss.

Round window coupling had the best hearing gain in air-conduction thresholds, and it was significantly better as compared to long process coupling, short process coupling, or stapes superstructure coupling. No deterioration of air-conduction thresholds has been reported in the studies evaluated.

### Bone-Conduction Devices (BCDs)

Compared to the Bonebridge® (Med EL, Innsbruck, Austria), the VSB has an increased bone-conduction amplification capacity.^13^, ^39^ The active transcutaneous devices are relatively new, with a small follow-up, mainly Osia. Lauren et al. published a follow-up study in patients older than 18 years of age with follow-up of up to 6 years with Bonebridge placed in the middle fossa, with no complications reported.^40^ In systematic reviews, both Bonebridge and Osia have shown a low complication rate.^41^

The authors of the studies included in this review do not explain the reasons for choosing VSB for those patients. But in patients with skull thickness below 3 mm and air conduction thresholds, VSB may be a preferable option instead bone conduction hearing devices. Although VSB surgery is more technically challenging, mainly due to the risk of facial nerve injury, it produces a more physiological stimulation only in the affected ear. It rarely causing skin problems that are common in percutaneous implants.^42^

### Facial nerve

Surgical injury of the facial nerve can be a major problem in patients with middle ear malformations. The absence of the external auditory canal causes the third portion to be displaced medially and anteriorly, increasing the risk of facial nerve injury during a mastoidectomy. This problem has been circumvented by intraoperative facial nerve monitoring and good surgical planning with the help of a high-resolution CT scan of the temporal bone. Few authors have reported significant problems in location the facial nerve trajectory. Wang et al. reported that the facial nerve concealed the round window in 11 of 16 patients studied, and the FMT was then placed on the stapes.^43^ Colletti et al.^17^ found facial nerve abnormalities in 58.3% of preoperative CT scans. Other authors placed the FMT in the round window via a retrofacial approach using an endoscope.^44^

### Complications

None of the studies reported significant postoperative complications. The main complications described associated with the facial nerve. The bone-conduction threshold changed slightly after surgery in a few patients who underwent surgery at the round window niche, where the risk of hearing loss is theoretically higher due to increased manipulation.^45^, ^46^ However, it should be noted that no randomized trials were available, and many studies were not included due to lack of standardization, which makes it difficult to conduct large studies in this field.

### Limitations

This review of the literature assessed hearing outcomes and postoperative complications in patients with middle and external ear malformations undergoing VSB surgery. The data presented a low level of scientific evidence. Significant heterogeneity among all data was found. Some studies presented only the mean values. Speech recognition scores, pre, and post air- conduction thresholds are the main audiological outcomes in almost all studies. Different speech analysis was not available.

The follow-up time was heterogeneous, ranging from 2 months to 6 years. Different from other studies, there was no report of device explantation or revision surgery, which may happen due to short follow-up time.

It should be noted that no randomized trials were available, and the lack of standardization makes it difficult to conduct large studies in this field.

## Conclusion

Bone conduction thresholds did not worsen in any studies included in the present review. VSB implantation resulted hearing gain of 40.5 dB of the air- conduction thresholds. The speech recognition index significant improvement with 86% or better scores. The worse the Jahrsdoerfer score, the greater the tendency to couple the FMT to the round window.

## Declaration of competing interest

The authors did not receive support from any organization for the submitted work. No funding was received to assist with the preparation of this manuscript.
